# Identifying pathogenicity of human variants via paralog-based yeast complementation

**DOI:** 10.1371/journal.pgen.1006779

**Published:** 2017-05-25

**Authors:** Fan Yang, Song Sun, Guihong Tan, Michael Costanzo, David E. Hill, Marc Vidal, Brenda J. Andrews, Charles Boone, Frederick P. Roth

**Affiliations:** 1 Donnelly Centre, Toronto, Ontario, Canada; 2 Department of Molecular Genetics, University of Toronto, Toronto, Ontario, Canada; 3 Department of Computer Science, University of Toronto, Toronto, Ontario, Canada; 4 Lunenfeld-Tanenbaum Research Institute, Sinai Health System, Toronto, Ontario, Canada; 5 Department of Medical Biochemistry and Microbiology, Uppsala University, Uppsala, Sweden; 6 Center for Cancer Systems Biology (CCSB), Dana- Farber Cancer Institute, Boston, Massachusetts, United States of America; 7 Department of Genetics, Harvard Medical School, Boston, Massachusetts, United States of America; 8 Canadian Institute for Advanced Research, Toronto, Ontario, Canada; HudsonAlpha Institute for Biotechnology, UNITED STATES

## Abstract

To better understand the health implications of personal genomes, we now face a largely unmet challenge to identify functional variants within disease-associated genes. Functional variants can be identified by trans-species complementation, e.g., by failure to rescue a yeast strain bearing a mutation in an orthologous human gene. Although orthologous complementation assays are powerful predictors of pathogenic variation, they are available for only a few percent of human disease genes. Here we systematically examine the question of whether complementation assays based on paralogy relationships can expand the number of human disease genes with functional variant detection assays. We tested over 1,000 paralogous human-yeast gene pairs for complementation, yielding 34 complementation relationships, of which 33 (97%) were novel. We found that paralog-based assays identified disease variants with success on par with that of orthology-based assays. Combining all homology-based assay results, we found that complementation can often identify pathogenic variants outside the homologous sequence region, presumably because of global effects on protein folding or stability. Within our search space, paralogy-based complementation more than doubled the number of human disease genes with a yeast-based complementation assay for disease variation.

## Author summary

Functional complementation assays of human disease-associated gene variants can reveal many more human disease variants at high confidence than current computational approaches, even using highly-diverged model organisms. However, this has generally only been possible for a minority of human disease genes for which orthologous complementation is known in the relevant model organism, so that alternative assays are urgently needed. Here we show that complementation relationships can be found for many additional human disease genes by exploiting paralogous human-yeast gene relationships, and that disease variant identification using paralogy-based assays performs on par with orthology-based assays.

## Introduction

As a result of rapid developments in sequencing technology, we are identifying many rare variants in individual human genomes [1]. To fully exploit this resource, we must be able to rapidly identify which of the many variants in each individual are most likely to be functional and disease-causing.

Yeast remains an extremely useful model organism for studying gene functions [2, 3], genetic interactions [4], protein-protein interactions [5–7], and genotype-phenotype relationships [8, 9]. The scale of experiments in yeast ranges from individual assays to high-throughput genome-wide experiments [10–12]. Of the ~6000 genes in yeast only about 15% are completely un-annotated with a function, and even for these genes there are clues from a wide range of large-scale experiments. Core cellular biology is well conserved between yeast and humans, with ~60% of yeast genes having human homologs and 87% of yeast protein domains being present in a human protein [13]. Functional complementation assays using model organisms can allow us to, for example, assess the functions of all possible missense variants of a gene in advance of their first appearance in the human population [14–16].

Assays of functional variation using complementation are constructed via two steps. First, a complementation relationship is identified, such that expression of a wild-type human gene product rescues phenotypic defects in a yeast strain lacking the cognate function. Second, the pathogenicity of genetic variants is assessed by comparing their ability to complement with that of the wild-type allele. Previously, we developed yeast-based functional complementation assays to evaluate the functional effects of missense variants in human disease-associated genes [17]. We and others have shown that yeast-based functional complementation assays can efficiently reveal the functionality of human genetic variants [15]. Indeed, our previous work showed that yeast-based functional complementation assays achieved three times the sensitivity of computational methods for detecting disease variants at the same high threshold of precision [17]. Although complementation assays have been largely restricted to orthologous human-yeast gene pairs, a few examples of paralogous complementation are known. For example, the human gene *RAC1* can complement the yeast *ras1*^−^ strain, suggesting functional similarity between these genes [18]. In another example, a recent systematic screen found that the human gene *SEC61A1*, implicated as a host factor for influenza, HIV and dengue viruses [19], can complement loss of the yeast gene *RFT1* [15]. Thus, the set of human disease genes with complementation-based functional variation assays could potentially be expanded via paralog relationships [18].

There are over 130,700 ‘disease-causing’ variants according to the most stringent annotation in the Human Gene Mutation Database (HGMD; as of November 2015), corresponding to 3535 unique disease genes [20]. Of these disease genes, 972 have an annotated ortholog in *Saccharomyces cerevisiae*, while another 762 genes have at least one paralog. (Except where noted, we follow the practical operating definition of “paralog” as any homolog not annotated as an ortholog.) If we include less stringent HGMD disease gene annotations (see Methods), the number of disease genes with a yeast ortholog rises to 1869, with an additional 1087 having a paralog.

Orthologs are (by definition) diverged by speciation rather than by duplication within a species, and it is generally believed that they are more likely to serve in the same biological role across species. In contrast, it is generally thought that paralogs—homologs that diverged by duplication within the genome of a species—are more likely to have evolved a distinct or specialized function. However, it is quite possible for orthologs to acquire different properties and for paralogs to retain the same function [21]. Although complementation assays based on human-yeast ortholog pairs can accurately predict pathogenic variants [17], it is unclear whether similar assays based on paralogs are as useful in predicting pathogenic variants. Therefore, we assessed the ability of paralogous complementation assays to detect pathogenic variation using an objective panel of disease and non-disease variants.

## Results

### Many complementation relationships exist for human-yeast paralogs

To expand the set of human disease genes with a functional complementation assay, we identified human disease genes, each having one or more essential yeast paralogs for which a conditional mutant was available. Because protein domains are distinct functional and structural units in a protein, because variants within a particular domain have a heightened chance of affecting structural and functional properties of the proteins in which they appear [22–24], and because domain-based mutational studies have proven useful in elucidating the functional and disease effects of variants [22, 25, 26], we also used protein domain annotations to select human-yeast paralogs for which all domains in the yeast protein could be found in the human protein. This yielded 314 human disease genes with a suitable yeast paralog to test. Given that a human gene may have multiple yeast paralogs, this resulted in a larger search space of 1060 human-yeast paralog pairs (S1 Table).

**Fig 1:**
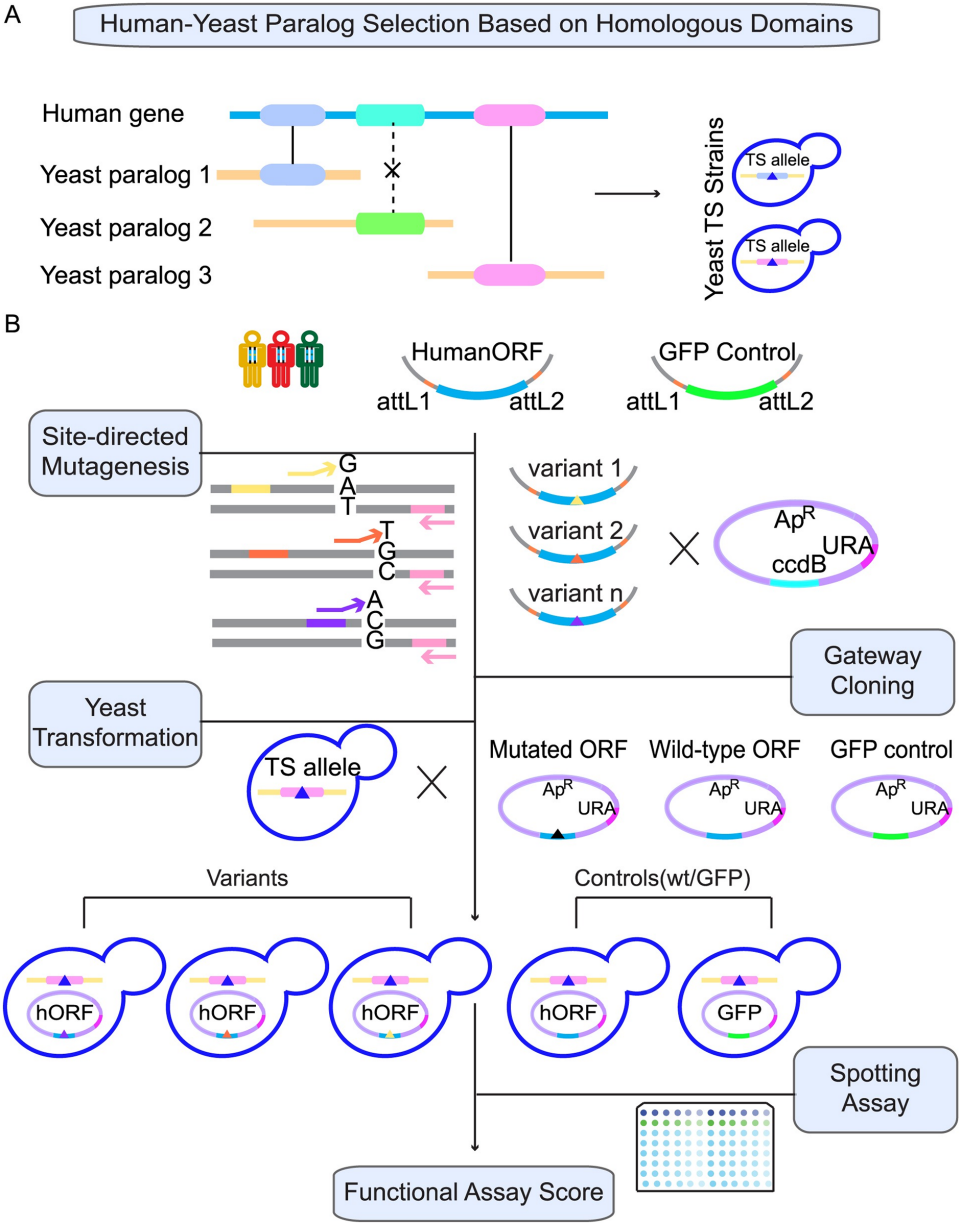
Schematic overview of process for assessing the functional effect of human disease-associated variants via complementation testing. A. We selected paralog pairs where a human disease protein has a yeast paralog for which all protein domains are also found in the human protein. Homologous pairs of domains are connected by solid lines, while non-homologous domain pairs are connected by a dashed line. B. For a subset of those paralog pairs for which we identified complementation relationships, we used these relationships to assess whether the functionality of variants in these assays predicted variant pathogenicity.

For each of the 314 human genes in our search space, we obtained an open reading frame (ORF) from the hORFeome 8.1 collection [7, 27], and generated a ‘humanized’ yeast expression plasmid via recombinational cloning [17]. To assess complementation for each human-yeast pair, the human protein was expressed in yeast strains bearing temperature-sensitive mutations [28] in the corresponding yeast gene, and growth was assessed at multiple temperatures (Fig 1; see Methods for detail).

In addition to functional complementation tests for 1060 human-yeast paralog pairs (including one positive-control paralog pair previously to complement), we assessed 7 additional known-complementing orthologous pairs as positive control. All complementation tests were performed twice, and 42 pairs yielded complementation in at least one replicate. These 42 included all 7 positive-control orthologous pairs, and 35 paralogous pairs. The 35 complementing paralog pairs included the positive control and 34 novel pairs, of which 33 were subsequently confirmed. (S1 Fig, see Methods for a complete description of complementation testing procedures.) Images showing negative functional assay results are available via the Dryad Digital Repository: http://dx.doi.org/10.5061/dryad.j05n0.

Thus, within a test space of 1060 human-yeast paralog pairs, we recovered 34 complementing pairs of which 33 (97%) were novel. Of the 314 human disease-associated genes tested, 33 (10.4%) yielded a complementation relationship with at least one yeast paralog.

### Some essential yeast genes are complemented by multiple human paralogs sharing only a single domain

**Fig 2:**
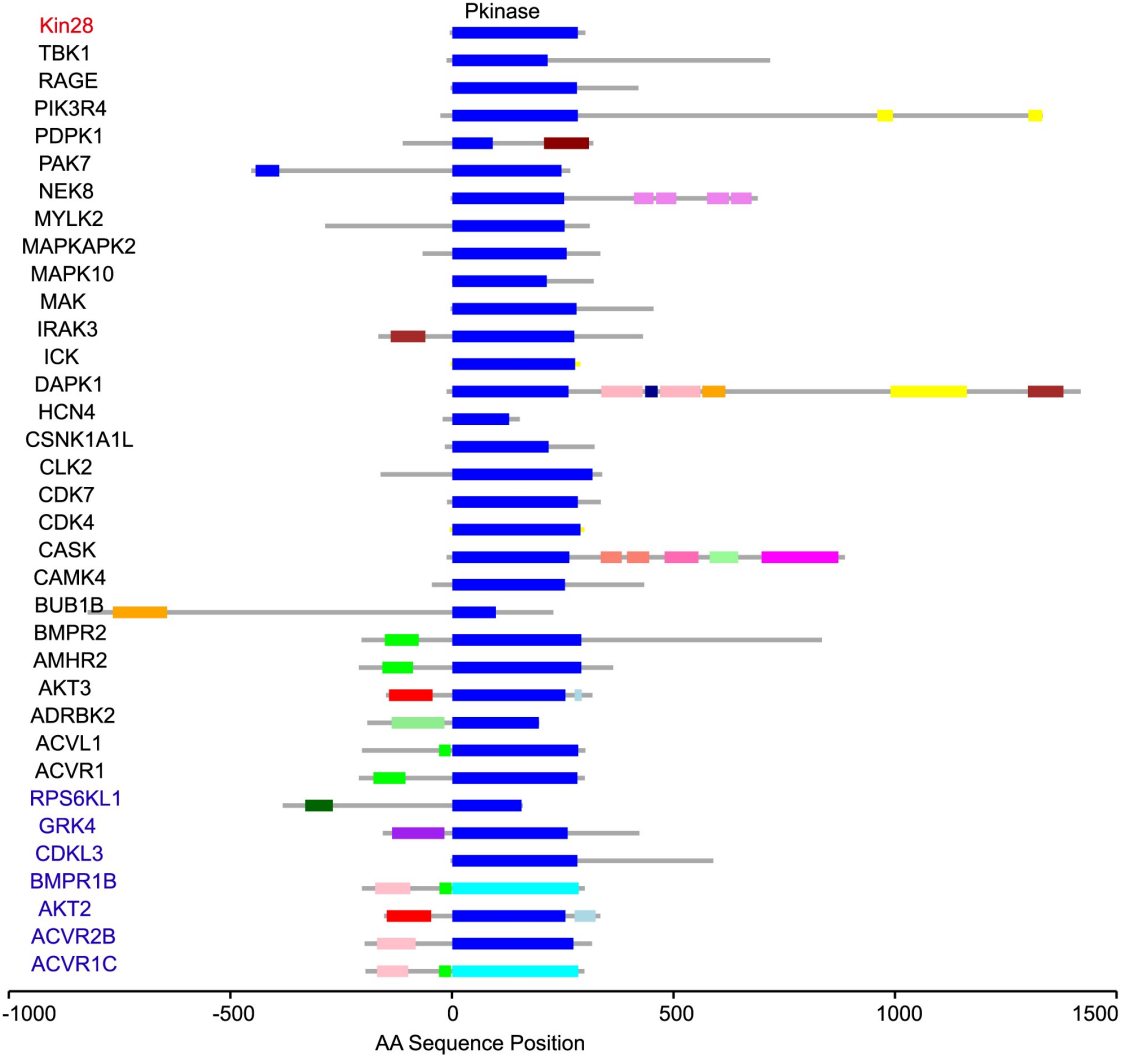
Protein domain architecture of yeast Kin28 and human paralogs. Shown are yeast Kin28 (red text), and human paralogs tested for complementation (in blue text if we found complementation and black text otherwise). Protein domain patterns Pkinase_Tyr (PFAM pattern PF07714) and Pkinase (PFAM pattern PF00069) are indicated in light and dark blue, respectively.

Among the 33 novel human-yeast paralog complementation assays established here, there were four yeast genes that could each be complemented by multiple human genes. For each of these yeast genes, the corresponding set of complementing human genes shared a common protein domain. For example, the function of yeast serine/threonine protein kinase Kin28 (ORF ID: YDL108W) could be complemented by expression of seven different human proteins (Fig 2): Ribosomal Protein S6 Kinase-Like 1 (RPS6KL1), G Protein-Coupled Receptor Kinase 4 (GRK4), Cyclin-Dependent Kinase-Like 3 (CDKL3), Bone Morphogenetic Protein Receptor, type IB (BMPR1B), V-Akt Murine Thymoma Viral Oncogene Homolog 2 (AKT2), Activin Receptor Type-2B (ACVR2B) and Activin A Receptor, Type 1C (ACVR1C), each sharing the same Pkinase protein domain found within yeast Kin28 (Table 1). However, each of these seven human proteins contain one or more additional protein domains and have different functions in different pathways. Indeed, the only apparent common thread among Kin28-complementing human proteins is the Pkinase protein domain.

**Table 1:** Seven human genes can complement yeast Kin28.

Human Gene Name	Human Gene Symbol	Protein Domain
Ribosomal Protein S6 Kinase-Like 1	RPS6KL1	PF00069,PF04212
G Protein-Coupled Receptor Kinase 4	GRK4	PF00069
Cyclin-Dependent Kinase-Like 3	CDKL3	PF00069
Bone Morphogenetic Protein Receptor	BMPR1B	PF00069,PF01064,PF08515
V-Akt Murine Thymoma Viral Oncogene Homolog 2	AKT2	PF00069,PF00169,PF00433
Activin Receptor Type-2B	ACVR2B	PF00069,PF01064
Activin A Receptor	ACVR1C	PF00069,PF01064,PF08515

**Fig 3:**
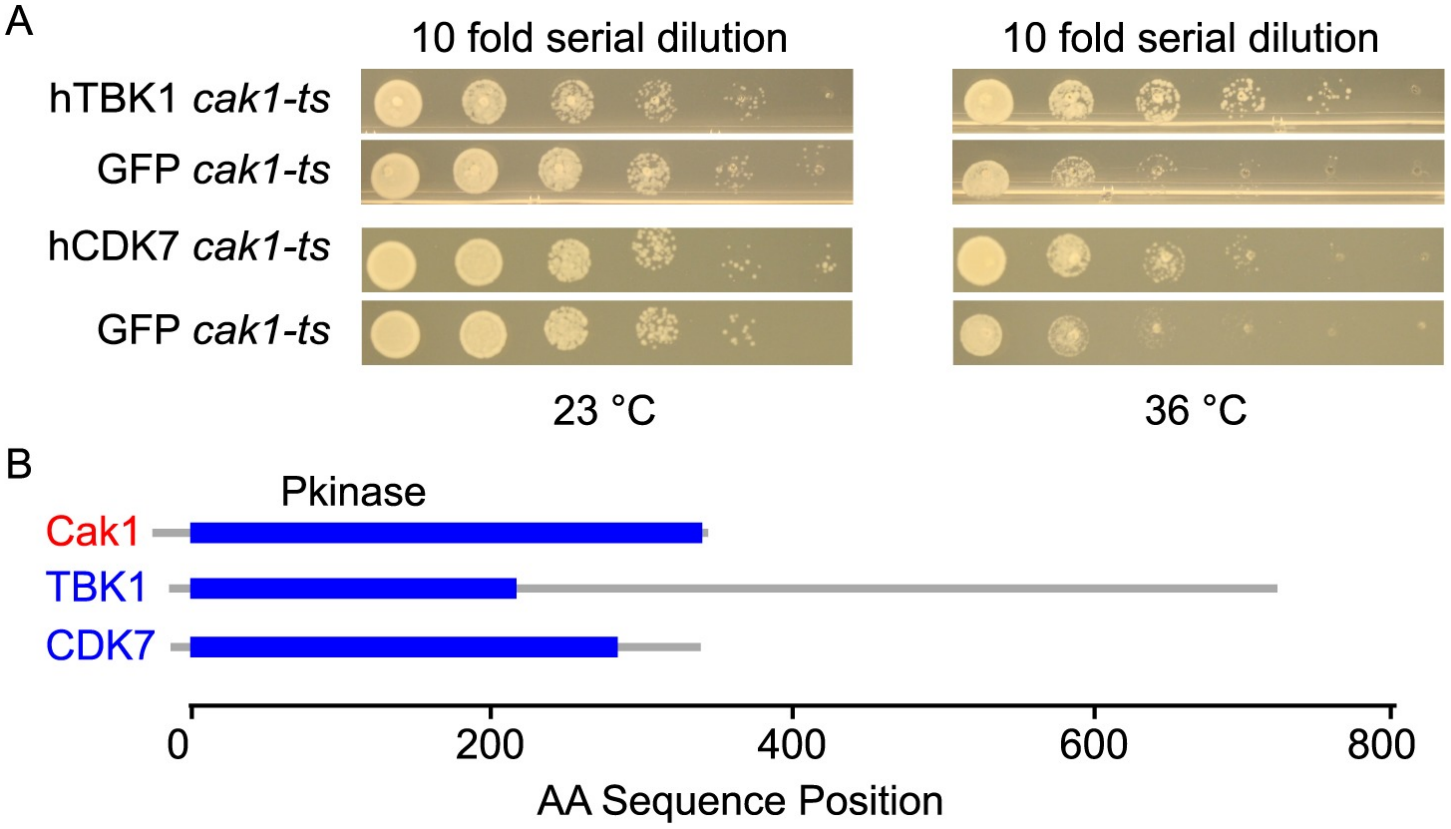
Functional assay and protein domain architecture of yeast Cak1 and its complementing human paralogs. (A) Functional complementation assay results showing that expression of human proteins TBK1 and CDK7 complements defects in a strain (YFL029C_tsa650) that encodes a temperature sensitive variant of Cak1 (described as “*cak1-ts*” above). (B) Pkinase domains are shown in dark blue. Complementing paralogs indicated in blue text.

The other three examples of yeast genes complemented by multiple human genes were *CAK1* (encoding Cdk-activating kinase Cak1), *SEC12* (encoding guanine nucleotide exchange factor Sec12), and *NAN1* (encoding Net1-Associated Nuclear protein Nan1). Complementing the loss of yeast Cak1 were two human genes encoding Serine/threonine-Protein Kinase (TBK1) and Cyclin-Dependent Kinase 7 (CDK7) (Fig 3), both of which contain a Pkinase domain. Complementing loss of yeast Sec12 were human genes *IFT122*, *ELP2*, and *GNB1L*, each sharing the WD40 repeat domain (PF00400). Loss of yeast Nan1 was rescued by human genes *PAFAH1B1* and *RFWD2*, also sharing the WD40 repeat domain. Thus, protein domain function, even when encoded by otherwise highly-diverged gene pairs, can be sufficiently conserved to allow functional rescue of a yeast protein and thus a potential assay for functional human variants.

Our search for complementation involved many kinases. Indeed, of the 1060 human/yeast gene pairs we tested, 480 (45%) of those pairs contained a yeast or human kinase-encoding gene. To understand this prevalence, we note that we only sought complementation where the human gene was a disease gene, where the yeast gene was essential, and where all domains in the yeast protein could be found in the human proteins. Although kinases do not seem to be enriched amongst human disease genes (they represent ~23% of annotated disease genes as compared with ~30% of non-disease-annotated genes), they are abundant. In yeast, there are 230 kinase-coding genes, of which 29 are essential. Moreover, 39% of human disease genes that had a yeast homolog are kinases, so that kinases are enriched for conservation in yeast. Thus, enrichment for kinases in our search space seems due to the fact that the kinase domain is ancient and found frequently in both yeast and human proteins. There were 14 yeast kinase-encoding genes and 56 human kinase-encoding genes amongst these 480 yeast/human kinase pairs, so that each gene appears in many pairs.

### Paralog complementation is only weakly predicted by sequence similarity

**Fig 4:**
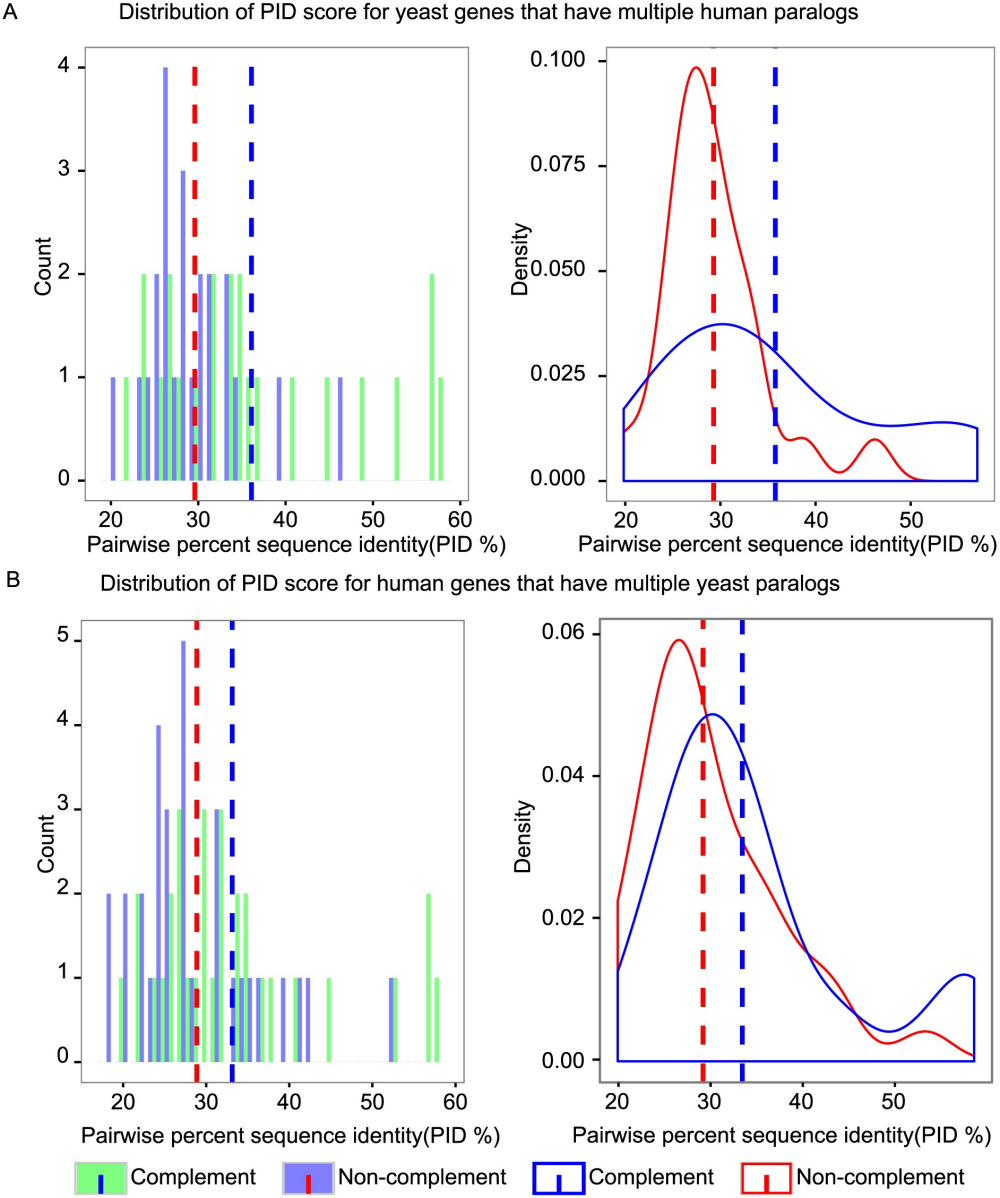
Relating sequence similarity and ability of a paralog to complement. The average percent identity (PID) score distribution is shown for human-yeast pairs such that multiple human paralogs were tested for a given yeast protein (A), and for human-yeast pairs such that multiple yeast paralogs were tested for a given human protein (B). In each case, the distribution is shown separately for complementing and non-complementing pairs. Each bin height is the count of human or yeast genes having a PID within the appropriate range for that bin. That complementing and non-complementing distributions are both shifted in positon relative to one another and highly overlapping suggests that sequence similarity is an informative but imperfect predictor of complementation.

We examined the extent of sequence identity between human disease-associated genes and their yeast paralogs. For each human and yeast gene pair, we calculated the pairwise sequence identity (PID; the percentage of aligned positions with identical residues). For a yeast gene with multiple human paralogs tested, we examined PID for complementing and non-complementing human-yeast paralog pairs. As expected, complementing pairs had higher PID than non-complementing pairs (Fig 4A, *P*-value = 0.007, Wilcoxon test). Similarly, for human genes that had multiple yeast paralogs tested, complementing pairs had relatively higher average PID (Fig 4B, *P*-value = 0.003, Wilcoxon test). A similar analysis performed for three additional sequence-identity calculation methods reached similar conclusions, except for one method which calculates a substantially lower percent identity in cases where the length of the aligned region differs greatly between two aligned proteins (see S1 File). Our results show that, as with human-yeast orthologs [12, 15], sequence similarity between human-yeast paralogs is correlated with—but only weakly predictive of—functional complementation. For example, a 30% PID threshold captured 60% of the complementing pairs, but 30% of non-complementing pairs also exceeded this threshold. Thus, systematic experimental testing will continue to be required for discovery of complementing paralog pairs.

### Assessing the pathogenicity of missense variants

Having established functional complementation relationships between human-yeast paralogs, we wondered whether these relationships could be exploited to assess the pathogenicity of human genetic variants. Of the 33 disease-associated genes for which we could identify a novel complementation relationship, there were 17 with known pathogenic missense variants according to HGMD DM annotation. To assess the ability of human/yeast paralog complementation assays to identify pathogenic variants (identified as those with high confidence “DM” annotation, indicating disease causality, from the HGMD database), we selected a subset of seven human disease-associated genes with multiple annotated disease-causing missense variants [29–31] (Table 2). Non-disease-annotated missense variants were present in the dbSNP database [32–34] for five of these seven genes. In total, we tested 19 disease-causing missense variants, each qualifying as causal according to the most stringent “DM” annotation in HGMD and the most stringent “pathogenic” annotation in ClinVar [35]. We also tested 16 non-disease-associated variants from dbSNP, selecting lower allele frequency variants where possible to better control for the generally low allele frequency of disease-causing variants.

**Table 2:** Deleteriousness predictions from functional complemention (FC), Polyphen-2 (PPH2) and PROVEAN.

Gene Symbol	Entrez	Variant	Disease Assoc?	FC Score	FC Prediction	FC Correct?	PPH2 Score	PPH2 Prediction	PPH2 Correct?	Provean Score	Provean Prediction	ProveanCorrect?	Within Aligned Region?
*CASK*	8573	T573I	No	0.6	Damaging	No	0.021	Neutral	Yes	-2.35	Neutral	Yes	No
*CASK*	8573	D471N	No	0.4	Neutral	**Yes**	0.005	Neutral	Yes	-1.48	Neutral	Yes	No
*CASK*	8573	M438L	No	0.4	Neutral	**Yes**	0	Neutral	Yes	-1.24	Neutral	Yes	Yes
*CASK*	8573	R430C	No	0.4	Neutral	**Yes**	0.035	Neutral	Yes	-2.51	Damaging	No	Yes
*CASK*	8573	R28L	Yes	0.8	Damaging	**Yes**	1	Damaging	Yes	-3.59	Damaging	Yes	Yes
*CYP19A1*	1588	M21T	No	0.6	Damaging	No	0.01	Neutral	Yes	-0.65	Neutral	Yes	No
*CYP19A1*	1588	M85R	Yes	0.8	Damaging	**Yes**	0.128	Neutral	No	-2.77	Damaging	Yes	Yes
*CYP19A1*	1588	W39R	No	0.4	Neutral	**Yes**	0.343	Neutral	Yes	-5.16	Damaging	No	Yes
*CYP19A1*	1588	M127R	Yes	0.8	Damaging	**Yes**	1	Damaging	Yes	-4.87	Damaging	Yes	Yes
*CYP19A1*	1588	Y81C	Yes	0.8	Damaging	**Yes**	1	Damaging	Yes	-6.87	Damaging	Yes	Yes
*DHDDS*	79947	K42E	Yes	0	Neutral	No	0.786	Damaging	Yes	-3.65	Damaging	Yes	Yes
*EMG1*	10436	D86G	Yes	0.6	Damaging	**Yes**	1	Damaging	Yes	-6.99	Damaging	Yes	Yes
*IFT122*	55764	G51A	No	0.2	Neutral	**Yes**	0.016	Neutral	Yes	-4.11	Damaging	No	No
*IFT122*	55764	T91I	No	0.2	Neutral	**Yes**	0.953	Damaging	No	-3.99	Damaging	No	No
*IFT122*	55764	S373F	Yes	0.6	Damaging	**Yes**	0.951	Damaging	Yes	-5.038	Damaging	Yes	No
*IFT122*	55764	L99W	No	0.4	Neutral	**Yes**	0.861	Damaging	No	-0.178	Neutral	Yes	No
*IFT122*	55764	R328W	No	0.2	Neutral	**Yes**	0.994	Damaging	No	-6.168	Damaging	No	No
*RAB33B*	83452	N148K	Yes	0.8	Damaging	**Yes**	0.005	Neutral	No	0.6	Neutral	No	No
*RAB33B*	83452	K46Q	Yes	0.8	Damaging	**Yes**	1	Damaging	Yes	-3.55	Damaging	Yes	Yes
*RAB33B*	83452	P142L	No	0.6	Damaging	No	1	Damaging	No	-9.99	Damaging	No	Yes
*RAB33B*	83452	T177M	No	0.6	Damaging	No	1	Damaging	No	-5.21	Damaging	No	Yes
*VCP*	7415	A232G	Yes	0.6	Damaging	**Yes**	0.005	Neutral	No	-1.87	Neutral	No	No
*VCP*	7415	I151V	Yes	0.4	Neutral	No	0	Neutral	No	-0.51	Neutral	No	Yes
*VCP*	7415	I27V	No	0.2	Neutral	**Yes**	0	Neutral	Yes	-0.43	Neutral	Yes	Yes
*VCP*	7415	Q19R	No	0.4	Neutral	**Yes**	0	Neutral	Yes	0.61	Neutral	Yes	Yes
*VCP*	7415	S171N	No	0	Neutral	**Yes**	0.004	Neutral	Yes	-1.18	Neutral	Yes	No
*VCP*	7415	T436I	No	0.4	Neutral	**Yes**	0.236	Neutral	Yes	-3.76	Damaging	No	No
*VCP*	7415	I206F	Yes	0.6	Damaging	**Yes**	0.983	Damaging	Yes	-3.7	Damaging	Yes	Yes
*VCP*	7415	L198W	Yes	0.6	Damaging	**Yes**	1	Damaging	Yes	-4.71	Damaging	Yes	Yes
*VCP*	7415	R159G	Yes	0.6	Damaging	**Yes**	1	Damaging	Yes	-6.56	Damaging	Yes	No
*VCP*	7415	R159C	Yes	0.8	Damaging	**Yes**	1	Damaging	Yes	-6.31	Damaging	Yes	No
*VCP*	7415	R159H	Yes	0.8	Damaging	**Yes**	0.517	Damaging	Yes	-2.97	Damaging	Yes	No
*VCP*	7415	R191G	Yes	0.6	Damaging	**Yes**	0.999	Damaging	Yes	-6.49	Damaging	Yes	Yes
*VCP*	7415	P137L	Yes	0.4	Neutral	No	1	Damaging	Yes	-9.31	Damaging	Yes	Yes
*VCP*	7415	R155G	Yes	0.4	Neutral	No	0.998	Damaging	Yes	-5.18	Damaging	Yes	No

The annotation of “FC correct?”, “PPH2 Correct?”, “Provean Correct?” is based on whether deleteriousness annotations from FC, PPH2 or Provean agree with current pathogenicity (HGMD “DM”) annotations. FC predictions that were correct according to HGMD “DM” are emphasized using a bold-text “Yes”.

For each of these 35 human variants, we generated an expression clone by site-directed mutagenesis and recombinational cloning, transformed it into the appropriate temperature-sensitive (TS) yeast strain, and assessed functional complementation (Fig 1; see Methods). For each genetic variant, this yielded a semi-quantitative Failure-to-Complement (FC) score, corresponding to the previously described “FCS score” [17]. FC scores were calibrated so that the positive (complementing) control wild-type human plasmid achieves a FC score of 0, and a Green Fluorescent Protein (GFP) negative (non-complementing) control achieves an FC score of 1. Following previous conventions, only variants with a score greater than 0.5 were considered deleterious [17, 36].

**Fig 5:**
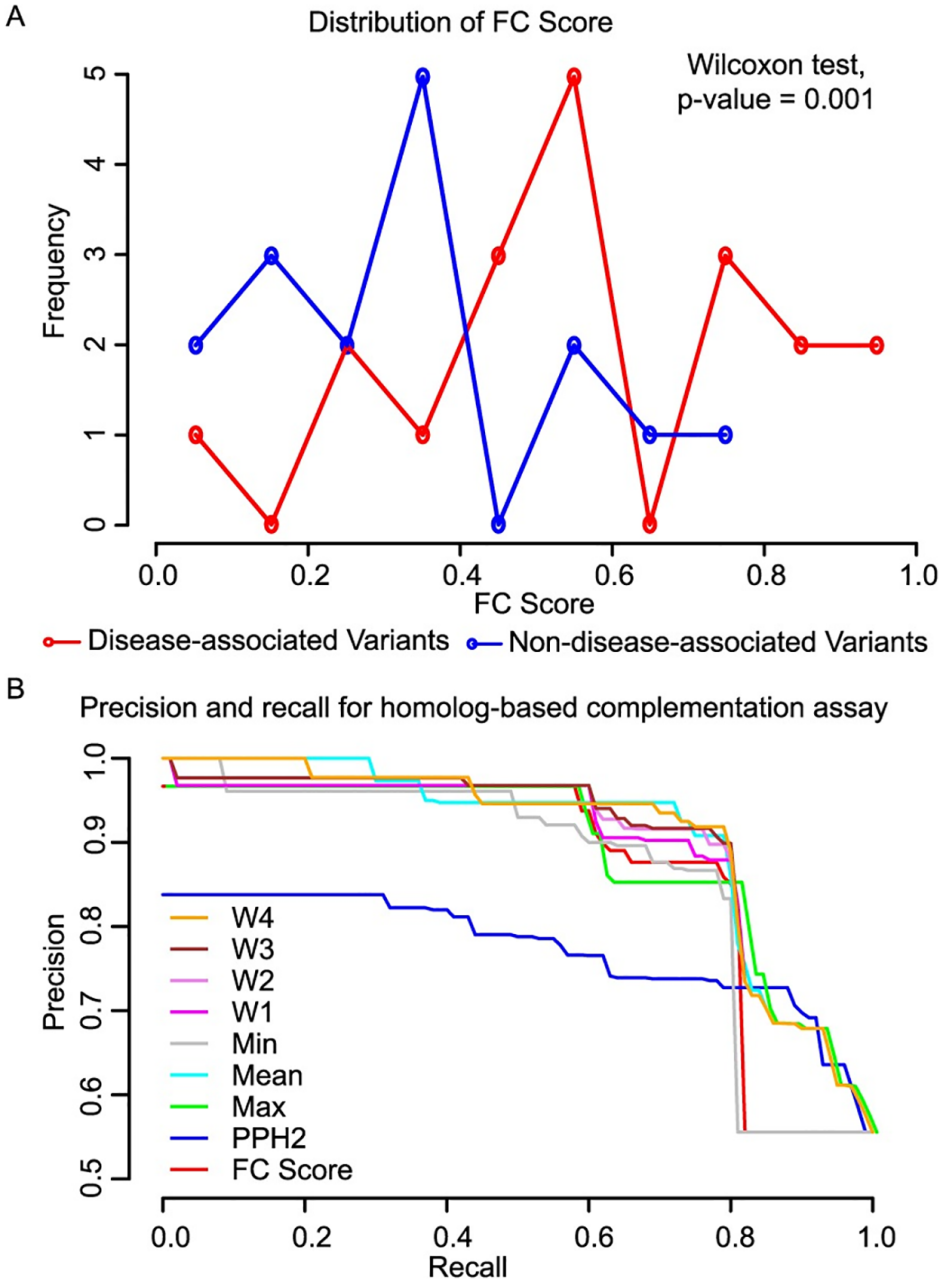
Ability of functional complementation to predict pathogenicity. (A) Distribution of FC scores for disease associated (red line) or non-disease-associated variants (blue line). FC scores from paralog-based complementation assays are significantly higher for disease-associated variants than non-disease-associated variants (*P*-value, Wilcoxon test). (B) Precision vs. recall performance for functional complementation scores (both paralog- and ortholog-based), PolyPhen-2 scores, and various options for combining the two approaches (see Methods).

Functional complementation assays predicted 15 (79%) of 19 disease variants and 4 (25%) of the 16 non-disease-associated variants we tested to be deleterious (S2 Fig). Our observation that 25% of non-disease-annotated variants failed to complement raises the possibility that many non-disease-annotated genetic variants may in fact impact gene function, so that our estimates of recall and precision may be conservatively low. Nevertheless, functional complementation assays clearly distinguish disease and non-disease-associated genetic variants: For the five genes that have both disease-associated and non-disease-associated variants, disease-associated variants exhibited significantly higher FC scores (*P*-value = 0.001, Wilcoxon test; Table 3, Fig 5A).

**Table 3:** Pathogenicity prediction performance for the human disease gene paralog test set.

Method	MCC	AUPRC	AUROC	REC90
PolyPhen-2	0.48	0.76	0.55	0.74
PROVEAN	0.37	0.7	0.52	0.71
Paralog-based FC	0.59	0.83	0.55	0.78

(MCC) Matthews correlation coefficient;

(AUPRC) area under the precision-recall curve;

(AUROC) area under the receiver-operating characteristic curve;

(REC90) recall at 90% precision.

Performance estimates for best-performing methods are indicated by underline

To put performance of functional complementation assays in the context of computational alternative methods, we applied PolyPhen-2 [36] and Protein Variation Effect Analyzer (PROVEAN) [37], two widely used computational methods for predicting pathogenic variants. At the 0.5 threshold, paralog-based functional complementation assays achieved 83% precision (fraction of predicted-deleterious variants that are annotated as pathogenic; 95% CI 58% - 96%) at 79% recall (fraction of pathogenic variants predicted to be deleterious). At a threshold score (0.5) which achieves the same 79% recall value, PolyPhen-2 achieved precision 75% (95% CI 51% - 90%). Different performance tradeoffs could be achieved at different thresholds. At the 0.7 threshold, paralog-based functional complementation assays achieved 100% precision (95% CI 60%-100%) at 42% recall. At the same 42% recall, PolyPhen-2 achieved 84% precision (95% CI 68%-100%). Although paralog-based variant testing numerically outperforms PolyPhen-2 in terms of precision at matched-recall thresholds, the limited sample sizes do not allow us conclude that this increase is significant. However, multiple performance measures—Matthews correlation coefficient (MCC), area under the precision-recall curve (AUPRC), area under the receiver operating characteristic (AUROC) curve, and recall at 90% precision (REC90)—suggest that paralog-based functional complementation assays are at least on par with computational methods in predicting pathogenicity (Table 3).

To more generally assess the performance of complementation-based pathogenicity assays against computational tests, we combined paralog-based and previous ortholog-based complementation pathogenicity tests [17]. At score thresholds where FC score and PolyPhen-2 both achieve a recall of 90%, the FC precision is 81% while PolyPhen-2 precision is 72%. Using the previously described performance threshold value of 0.5 for the FC score [17] achieves a recall of 78% and precision of 89% for the FC score. At a matched 78%, recall threshold, PolyPhen-2 yields a lower precision of 73% (Fisher’s exact test *P*-value = 0.003). A similar comparison using only ortholog-based assays yielded the same conclusion, albeit with a less significant *P*-value of 0.008 [17]. Thus, inclusion of paralog-based complementation strengthens previous conclusions that complementation-based identification of functional variation outperforms current computational approaches.

We next investigated whether the combination of FC with PolyPhen-2 scores could yield performance that exceeds either approach alone. We used seven alternative ways to combine these scores: minimum, maximum, mean, and four alternative weighted means (w1 through w4) (Fig 5B). The results confirmed our previous conclusion that combining FC and PolyPhen-2 scores can improve the performance in the high precision/low recall region.

**Fig 6:**
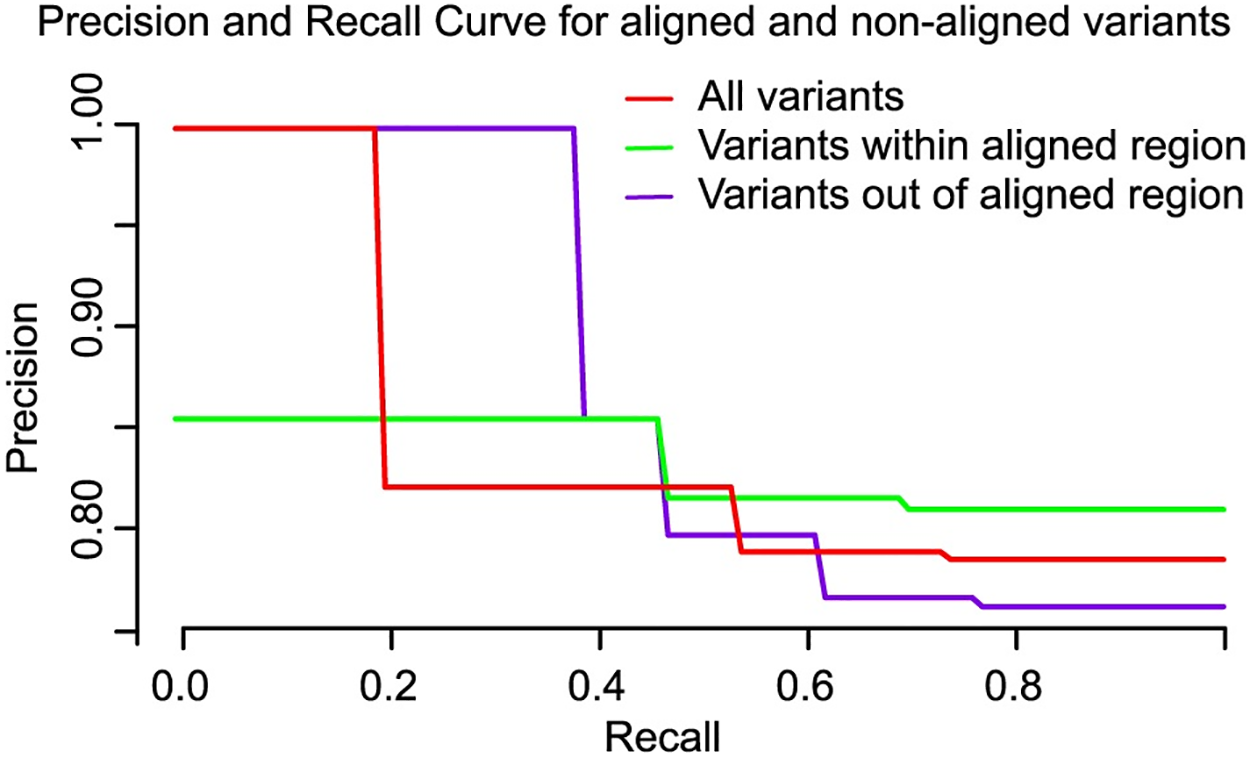
Performance of pathogenic variant identification does not strongly depend on whether the variant is in the aligned region. Here we show precision vs recall performance for varants that either do (‘aligned’) or do not (non-aligned) fall within the sequence region that can be aligned between human and yeast homologs.

We wondered whether complementation assays are capable of detecting pathogenic variants when these variants fall outside of the aligned homology region. It is possible that variants will affect additional human gene functions that are not needed for complementation, so that such pathogenic variants will be missed. However, variants which alter protein folding, or stability in a human cell may often do the same in a yeast cell. Interestingly, the ability of complementation to identify disease variation did not depend strongly on whether or not the variation falls within the aligned region of homology between yeast and human genes. As shown in Fig 6. at a score threshold achieving 90% recall, the likelihood of detecting a disease variant was comparable: 0.76 and 0.87 respectively for variants inside and outside of the aligned region of human and yeast paralogous pairs. Taking ortholog- and paralog-based complementation assay data together, the distributions of FC scores for variants inside and outside of the aligned region were statistically indistinguishable (*P*-value = 0.37, Wilcoxon test). All Wilcoxon tests are unaffected by our somewhat arbitrary assignment of numeric FC scores to different qualitative classes of observed complementation, because these tests only use the ranking order of quantitative values. At score thresholds yielding a recall of 90%, pathogenic variant detection variation achieved a precision rate of 92% and 88% respectively for variants inside and outside of the aligned region. Thus, functional complementation assays are capable of accurately detecting pathogenic variants, even outside of the aligned homology region.

## Discussion

Considerable effort has been made to understand how genetic changes give rise to the molecular effects that cause diseases [38–40]. There are many databases and tools for prioritizing candidate single nucleotide polymorphisms (SNPs) or hypothesizing the molecular causes of genetic disease. Functional complementation assays enable identification of pathogenic disease variants with substantially greater sensitivity than computational methods [17]. Although previous trans-species functional complementation assays have been almost exclusively based on orthology relationships, our systematic search yielded novel paralogy-based functional complementation assays for 33 human disease genes.

The gene *RAB33B*, which encodes a small GTP-binding protein of the RAB family and is associated with Smith-McCort Dysplasia, can illustrate paralog-based functional complementation. We successfully observed failure to complement for the two disease associated variants, P219S and K46Q [41, 42]. Interestingly, both non-disease-annotated variants, P142L (rs369719131) and T177M (rs140381459), also showed loss of complementation. Our findings agreed with PolyPhen-2 and PROVEAN which each also predicted them to be deleterious. All four variants tested are within the Ras domain. Thus, even though variants P142L and T177M are not known to be associated with disease, they appear to affect protein function.

Another example is the human *CASK* gene, which encodes calcium/calmodulin-dependent serine protein kinase. *CASK* encodes a 921-amino acid polypeptide with an N-terminal calcium/calmodulin-dependent protein kinase-like domain, PDZ and SH3 domains, a potential protein-binding motif, and a domain homologous to guanylate kinase [43]. Sequence variants in CASK cause intellectual disability [44]. The only annotated disease variant we tested in CASK was the kinase domain variant R28L causing FG Syndrome [45], an X-linked disorder causing intellectual disability, physical anomalies and developmental delays. This variant exhibited loss of complementation. We also tested several non-disease-associated CASK variants (D471N, M438L, R430C, and T573I). Three of the four non-disease variants tested retained the ability to complement. By contrast, the variant T573I (rs141840001), despite not being annotated as associated with Mendelian disease [30, 35, 46] or via any GWA study [47], showed reduced complementation. This variant was originally identified in a clinical genetics laboratory (Emory Genetics Laboratory, ClinVar accession RCV000175306.1) in an autistic male, so that the evidence of functionality we found for T573I may spur further investigation.

In addition to yielding a direct benefit in the form of novel functional assays, our systematic search for paralogous complementation enabled some general observations about complementation relationships. First, as with orthologs, sequence similarity is only a very weak predictor of complementation relationships (Fig 4), necessitating experimentation to identify complementation relationships.

**Fig 7:**
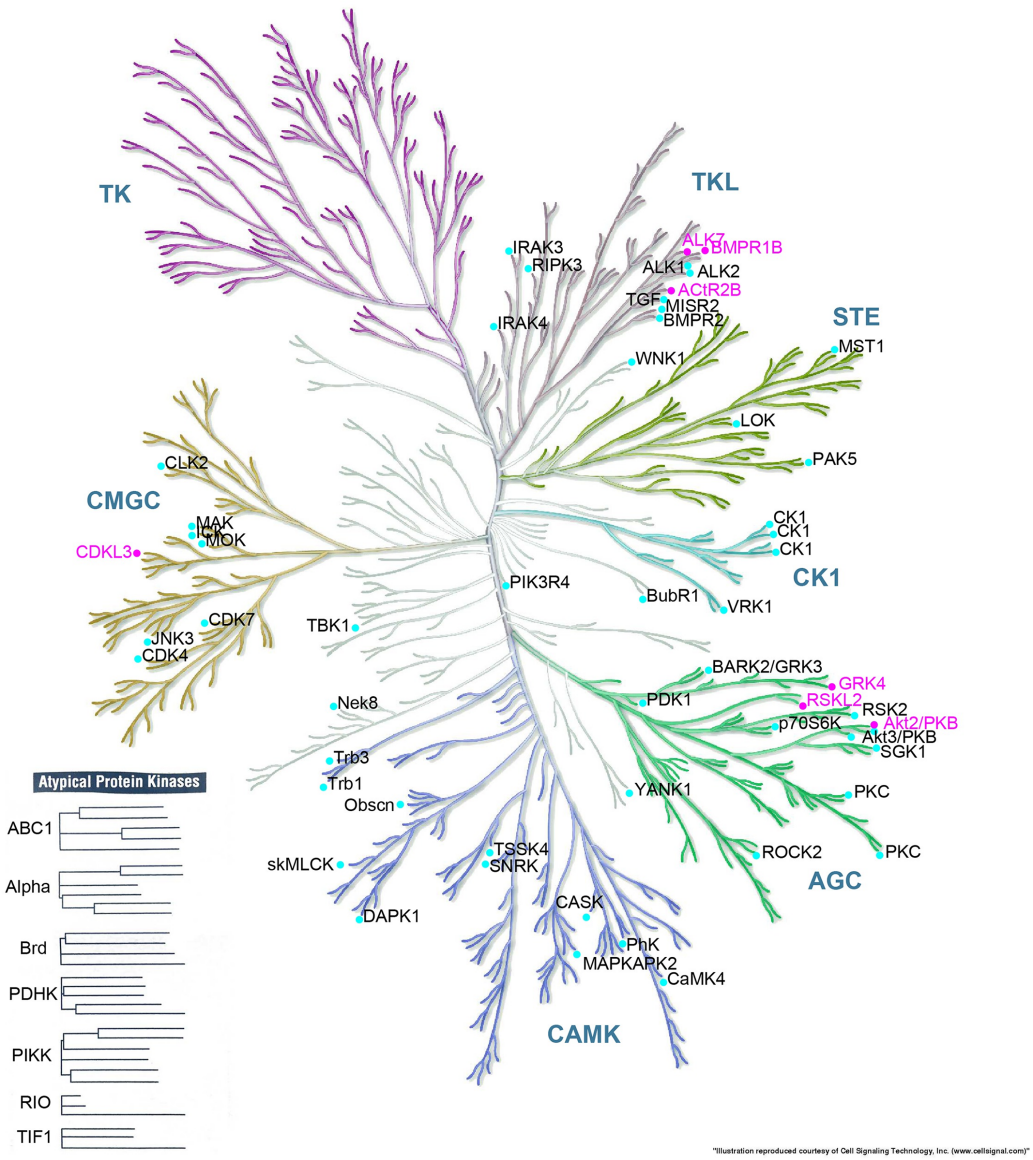
The kinome tree of yeast Kin28 and its kinase paralogs tested here. Kinases that can complement yeast Kin 28 were colored in pink, other kinases tested for ability to complement yeast Kin28 were colored in cyan. (The image was generated from the Kinome-Render Tool [49] hosted at Cell Signaling, Inc.).

Second, despite the idea that paralogs often have divergent functions, we found that multiple human genes (having in common a single protein domain) can sometimes complement the same yeast gene. For example, the seven human disease-associated genes that can complement yeast *kin28* all encode a protein kinase domain. Interestingly, the seven complementing genes fall into three different major kinase groups, including TKL kinases, CMGC kinases and AGC kinases (Fig 7). An additional 31 human disease-associated genes that encode the same protein domain (many of which fall into the same three major kinase groups) did not complement yeast *kin28*. Using the multiple sequence alignment tool Clustal [48] to examine the phylogenetic tree of tested human protein homologs of yeast Kin28), we also found no evident clustering of the yeast Kin28-complementing human homologs that could distinguish them from non-complementing human kinases. This result highlights the idea that closer evolutionary relationships do not guarantee complementation. When we mapped the 38 kinases to KEGG and REACTOME pathways, 5 of 7 complementing kinases mapped to signal transduction pathways, but this was not significantly different from rate at which 22 tested non-complementing kinases of 31 mapped to the same pathway (P = 0.6, Fisher’s exact test). Thus, we found no obvious predictors of which kinases were more likely to complement (S4 Table).

We were surprised to find that the success of paralog-based complementation assays is on par with ortholog-based complementation in terms of identifying disease variation. Examining a test set of disease- and non-disease-associated variation, we found that paralog-based complementation could detect ~78% of pathogenic variants at 90% precision, which was statistically indistinguishable from the performance of ortholog-based complementation. The combination of paralog and ortholog-based complementation continues to outperform current computational approaches.

Paralog-based assays have high potential to extend the arsenal of assays to assess the functionality of human coding variation. This is despite the fact that paralogs yielded a complementation relationship for a smaller fraction of human genes than had been observed for ortholog pairs: this fraction was 10% in the current study as compared with 19% for ortholog pairs [17]. Indeed, Kachroo *et al* [12] achieved an even higher 47% rate of complementation for a subset of orthologous pairs that are “one to one”, i.e., for which there are no close paralogs in either human or yeast. According to the YeastMine database [50] there are 773 additional human disease-associated genes with yeast paralogs, suggesting that a functional assay could potentially be developed for at least ~70 additional human disease-associated genes through further examination of paralog complementation. According to HGMD, about 3019 human disease-associated genes have paralogs in either *S*. *cerevisiae* or *Schizosaccharomyces pombe*. Simple extrapolation suggests that a more exhaustive search for complementation relationships in these two yeast species could yield complementation assays for assessing functional variation in 300 human disease genes. Considering multicellular model organisms, the number of potential complementation assays increases further (see Table 4 for a summary of human disease-associated genes with either an ortholog or paralog in five model animal systems). Given that complementation tests work as well as they do for identifying pathogenic variation in the billion-year diverged model organism *S*. *cerevisiae*, it stands to reason that other model systems (including complementation in human cells where cell-autonomous selectable phenotypes are known) should also be explored.

**Table 4:** Numbers of human disease-associated genes with orthologs and paralogs in five model species.

Organism	Human disease-associated genes	
	Orthologs	Paralogs
*S*. *cerevisiae* or *Schizosaccharomyces pombe*	6648	3019
*Mus musculus*	5547	256
*Rattus norvegicus*	5492	265
*Danio rerio*	4619	231
*Drosophila melanogaster*	3021	384*
*Caenorhabditis elegans*	2665	169

*This figure is conservative, in that the HGMD source for this information used a more stringent criterion for paralogy (elsewhere in this study homologs without annotated orthology are referred to as paralogs).

Our results combining paralog- and ortholog-based complementation tests show that these assays can be used to accurately identify pathogenic variants even when those variants fall outside of the aligned region. This is consistent with the idea that many deleterious variants affect protein folding or stability and disrupt the function of the entire protein. Thus, even where only a single domain is required for a human protein to complement its yeast paralog, that relationship can be exploited to detect a substantial subset of functional variation throughout the length of the human protein.

It is worth revisiting our working definition of paralogy (homology without annotated orthology). Paralogs under this definition may be previously unrecognized orthologs, and gene pairs with complementation relationships may be enriched in such cases. However, for the practical purpose of identifying pathogenic variants using a complementation assay, it seems that the distinction between paralogy and cryptic orthology is essentially irrelevant. In either case, complementation relationships between human genes and their homologs in other species beyond *S*. *cerevisiae* provide substantial further opportunities to study the functional properties of human disease-associated variants.

One potential limitation of complementation testing is that, while it may accurately detect many loss-of-function variants, we expect that it is less likely to identify gain of function variants. We reviewed the primary literature for the 19 disease variants we tested via paralog-based complementation. Of these 19, the literature suggested “loss of function” for 16 and “gain of function” for only one (S3 Table). The putative “gain of function” variant retained its ability to complement, and was thus, as expected, not detected as damaging by our complementation assay.

Given that computational approaches are faster, cheaper and available for a wider range of genes than are functional complementation assays, it is worth asking whether systematic experimental variant assessment is worth pursuing. Where variant assessment is critical for diagnosis and therapy, and where computational methods cannot return a sufficiently confident call for a large fraction of disease variants, alternatives are clearly needed. Moreover, new advances in “deep mutational scanning” have enabled the *en masse* application of a cell-based functional assay to essentially all missense variants for a given protein [51]. For a fixed initial cost, deep mutational scans can provide a comprehensive ‘look-up’ table allowing instantaneous interpretation of missense variants as they appear in the clinic.

## Materials and methods

### Selecting human-yeast homologs for testing

To systematically test the ability of wild-type human disease-associated genes to rescue mutations in paralogous yeast genes, we defined the search space to be human genes for which HGMD [29–31] has annotated one or more alleles as being ‘DM’ (disease-causing) and for which a clone was available in ORFeome version 8.1 [27].

Because protein domains are distinct functional and structural units in a protein, because variants within a particular domain have a heightened chance of affecting structural and functional properties of the proteins in which they appear [22–24], and because domain-based mutational studies have proven useful in elucidating the functional and disease effects of variants [22, 25, 26], we also used protein domain annotations as a criterion for selecting human-yeast paralogs. We searched both yeast and human genes against the Pfam domain types from the Pfam protein domain family database (version 27) [52], using an *E*-value cutoff of 0.001 [53], and identified cases where all protein domains encoded by a yeast gene were fully ‘covered’ by a human gene. In our previous study [17], we used the InParanoid database [54] to select yeast/human orthologous pairs for which the human gene had at least one disease-associated variant according to either HGMD or OMIM databases. Here yeast/human pairs were chosen similarly, except that we accepted all homologs reported by InParanoid except those annotated as orthologs. The InParanoid program uses NCBI-BlastP pairwise similarity scores for constructing orthology groups. An orthology group is initially composed of two so-called seed orthologs that are found by mutual best hits between two proteomes.

Considering only paralog pairs where the yeast gene was essential and had an available temperature sensitive mutation, where the human gene had an available expression clone, and where all protein domains in the yeast gene were covered in the corresponding human gene, we selected 1060 human-yeast paralog pairs corresponding to 314 human genes and 162 yeast genes. We note that a single gene in one species can have multiple paralogs in another species, and thus appear in multiple tested paralog pairs.

### Constructing wild-type human ORFs and human ORFs with disease-associated variants

Wild-type human disease-associated ORFs were selected from the human ORFeome version 8.1 [27]. As described in Sun *et al* [17], human ORFs with disease-associated variants were constructed by site-directed mutagenesis using the Thermo Scientific Phusion Site-Directed Mutagenesis Kit. The Gateway Donor plasmid was amplified using phosphorylated primers that introduce the desired changes followed by a 5-minute, room-temperature ligation reaction. The resulting plasmid was then transformed into NEB5α competent *E*. *coli* cells (New England Biolabs).

### Constructing the *S*. *cerevisiae* expression plasmid pHYC-URA-ORF/GFP

All expressed ORFs used in these studies—including wild-type human disease-associated ORFs, human ORFs with constructed alleles, and the GFP control—were transferred into the destination vector pCM188- URA [55] by Gateway LR reactions using the All Gateway LR Clonase enzyme kit from Life Technologies. The destination vector pCM188-URA was obtained from ATCC, and subsequently altered to be Gateway compatible following the procedure applied in Sun et al (Genome Research 2016) to vectors pHYCDest-LEU2 and pHYCDest-NatMX. Plasmids generated by Gateway LR cloning were transformed into NEB5α competent *E*. *coli* cells (New England Biolabs) and selected on LB Agar plates with 100μg/mL Ampicillin. All plasmid DNA samples were isolated and purified using the NucleoSpin 96 Plasmid toolkit (Ref: 740625.24) and confirmed by Sanger sequencing. Plasmids carrying expressed ORFs were then transformed into the corresponding yeast temperature-sensitive strains.

### Yeast-based functional complementation assay

Yeast temperature-sensitive (TS) strains carrying human ORFs or GFP control were spotted in a 10-fold dilution series and grown at a range of temperatures (room temperature of ~24°C, and 28, 30, 32, 33, 34, 35, 36 and 38°C). Results were interpreted by comparing the growth difference between the yeast strains expressing human genes and the corresponding control strain expressing the GFP gene. Each test was initially performed twice and pairs were found in at least one replicate were considered complementation candidates. For confirmation experiments, we went back to the glycerol stock of the relevant yeast TS strain, and re-transformed the expression plasmid for the candidate complementing human gene (and negative GFP control) into this fresh isolate. We further considered only those candidates passing a third replicate functional complementation assay.

### Predicting functional effects for missense variants

To predict functional effects for each missense genetic variant, we assessed complementation with the above-described yeast spotting assays and assigned a semi-quantitative Failure-to-Complement (FC) score (corresponding to the previously-described FCS score [17]). Semi-quantitative FC scores were assigned to each variant: 0 (wild-type-like complementation), 0.6 (reduced complementation), 0.8 (severely reduced complementation) and 1 (complete loss of complementation). The predicted functional impact score for disease-associated variants were generated by the two best-performing computational methods in our previous study [17]: Polymorphism Phenotyping v2 (PolyPhen-2 [36, 56]) and PROVEAN [37].

As a pre-processing step before combining computational and FC scores, we followed the same method introduced by Sun *et al*, to calibrate each scoring system. To calculate the calibrated score for each disease variant, we combined the variants tested in both paralog-based and ortholog-based complementation assays, and randomly separated them into 10 groups. Precision within the FC (or PolyPhen-2) training data was calculated at different thresholds of each scoring method. precision and recall performance was then evaluated for seven methods of combining the two scores: minimum, maximum, mean, and four alternative weighted mean methods, where each method takes the form of α × calibrated-FC-score + (1- α) × calibrated-PolyPhen2-score. Specifically, methods w1, w2, w3 and w4 corresponded to α values of 0.9, 0.8, 0.7 and 0.6.

The area under the precision-recall curve (AUPRC) was calculated using R package “PRROC”. When comparing the performance of functional complementation assays in predicting disease associated variants in either aligned or not aligned regions, we wished to account for the fact that changing the prior probability of pathogenicity can alter precision estimates. Therefore, performance was estimated using the ratio of AUPRC relative to the prior probability (designated as AUPRC_norm) instead of AUPRC.

## Supporting information

S1 Table Human-yeast complementing paralogous pairs. (XLSX)

S2 Table 1060 Human-yeast homologous pairs tested. (XLSX)

S3 Table 35 human disease variants tested with FC Score. (LOF: loss of function, GOF: gain of function).
(XLSX)

S4 Table Pathway information of human kinases which are paralogs of yeast Kin28. (XLSX)

S1 Fig Functional assay result of complementing paralogous pairs at permissive, semi-permissive and non-permissive temperatures. (PNG)

S2 Fig Functional assay result and FC score of human variants at permissive, semi-permissive and non-permissive temperatures. (PNG)

S1 File Testing association between sequence similarity and complementation relationships for human-yeast paralogs. (DOCX)
