# Supplementary figures and images for: Identifying pathogenicity of human variants via paralog-based yeast complementation
Source: PLoS Genet. 2017 May 25;13(5):e1006779. doi: 10.1371/journal.pgen.1006779 (PMC5466341; doi:10.1371/journal.pgen.1006779)

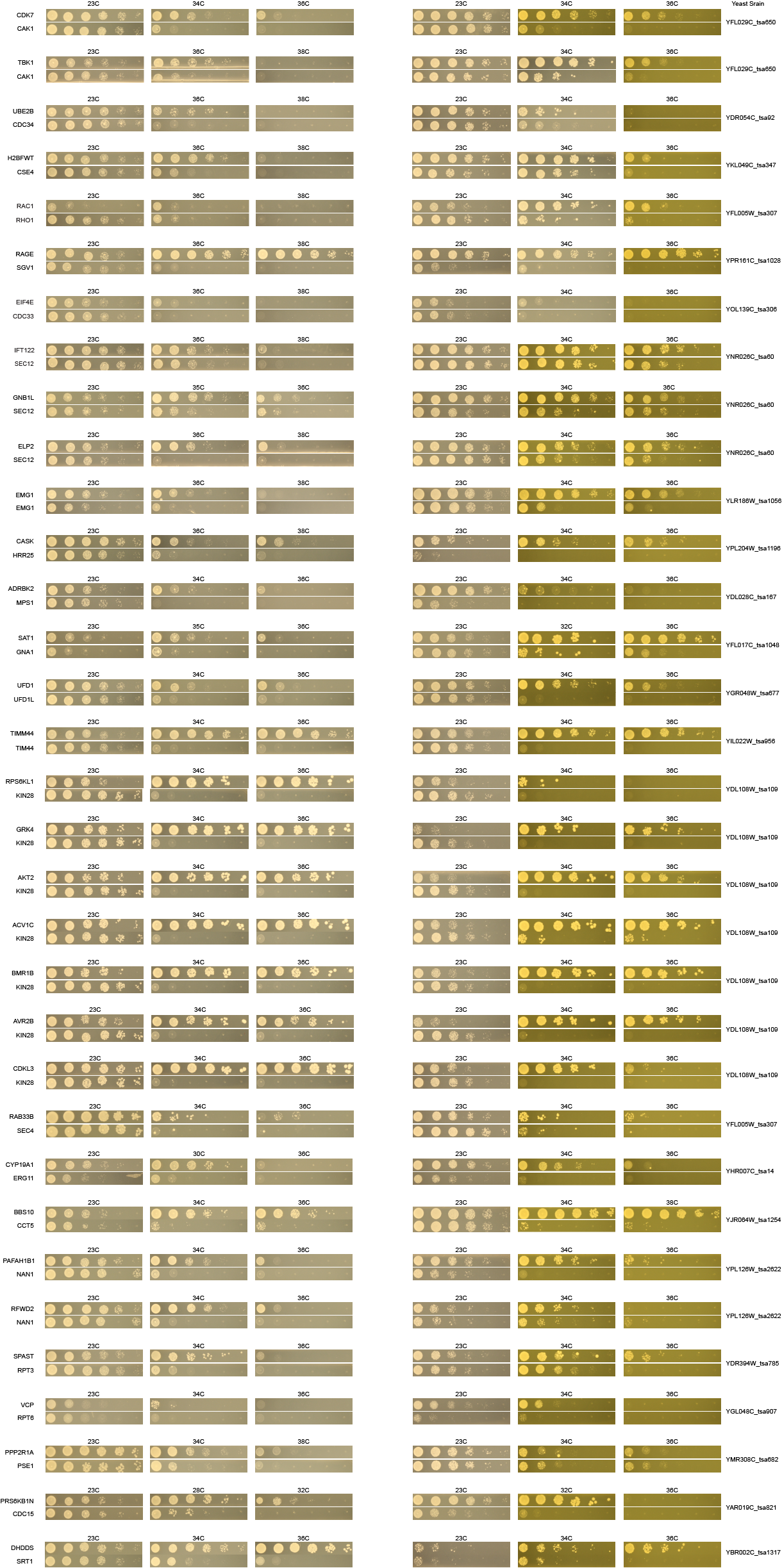

Supplement: S1 Fig — (PNG) [file pgen.1006779.s005.png]

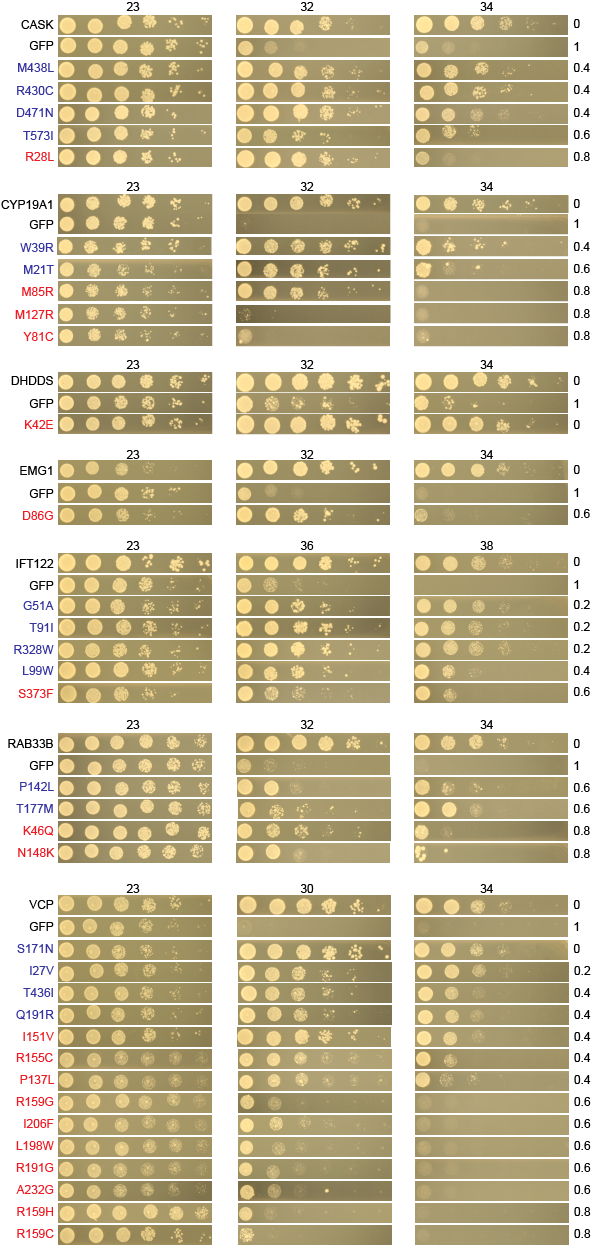

Supplement: S2 Fig — (PNG) [file pgen.1006779.s006.png]
