# Supplementary material for: Identifying pathogenicity of human variants via paralog-based yeast complementation
Source: PLoS Genet. 2017 May 25;13(5):e1006779. doi: 10.1371/journal.pgen.1006779 (PMC5466341; doi:10.1371/journal.pgen.1006779)
Supplement: S1 File — (DOCX) [file pgen.1006779.s007.docx]

**Testing association between sequence similarity and complementation relationships for human-yeast paralogs.** In addition to the percent sequence identity (PID) score used in the main text, which was defined as the percentage of aligned positions with identical residues, we examined three other sequence-identity calculation methods. The PID_ai_ score was defined as the ratio of number of identical aligned positions relative to the sum of aligned and internal-gap positions. The PID_sl_ score was defined by the ratio of number of identical positions to the total number of position in the shorter sequence, while PID_al_ was defined by the ratio of number of identical positions to the average length of the human and yeast gene pairs. Regardless of which sequence-identity calculation method was used, where yeast genes having more than one human paralog were tested, the paralogous pairs that complemented showed higher sequence identity (*P*-value = 0.0072 for PID; *P*-value = 0.0089 for PID_ai_; *P*-value = 0.0037 for PID_sl_ ; *P*-value = 0.0046 for PID_al_; Wilcoxon Test). For human genes having multiple yeast paralogs, those paralogous pairs that complemented showed higher sequence identity in all cases except PID_ai_ which considered both the aligned positions and internal gaps (*P*-value = 0.0034 for PID; *P*-value =0.1995 for PID_ai_; *P*-value = 0.0090 for PID_sl_; *P*-value = 0.02 for PID_al_; Wilcoxon Test). Differences between results based on PID_ai_ and PID scores are consistent with the observation that human genes tend to be longer than their yeast counterparts.
